# Plasma Aβ42/Aβ40 and phospho‐tau217 concentration ratios increase the accuracy of amyloid PET classification in preclinical Alzheimer’s disease

**DOI:** 10.1002/alz.090434

**Published:** 2025-01-03

**Authors:** Robert A Rissman

**Affiliations:** ^1^ University of California San Diego, La Jolla, CA USA

## Abstract

**Background:**

Our data from several clinical trials of individuals with asymptomatic AD demonstrates that plasma Aβ42/40 quantification by mass spectrometry can serve as a reliable biomarker for predicting elevated brain amyloid as detected by PET. We investigated how adding plasma p‐tau measures to our plasma Aβ42/40 algorithm to streamline identification of eligible participants and reduce burden and trial cost. To determine if the addition of plasma p‐tau181 and/or p‐tau217 concentrations can improve plasma Aβ42/40 algorithms to correctly identify participants with amyloid burden of >20 centiloids with the NAV4694 tracer among individuals screening for participation in the AHEAD preclinical AD trial.

**Method:**

Plasma amyloid and tau were measured using C_2_N Diagnostics mass spectrometry. Participant samples (N = 1085) collected prior to the incorporation of plasma Aβ42/40 testing during screening were used. Plasma samples consisted of those with adequate amyloid PET levels (n = 364; 33%) to be eligible for AHEAD and those who did not and screen failed (n = 747; 67%). We quantified Aβ (Aβ42/40) and tau species, including the phosphorylated (p‐tau) and non‐phosphorylated (np‐tau) forms of tau181 and tau217. A ratio of p‐tau to np‐tau was calculated (p‐tau181r and p‐tau217r) to normalize inter‐individual differences in np‐tau. We conducted Receiver Operating Characteristic (ROC) curve analyses fagainst amyloid PET status (>20 centiloids). We fit a Mixture of Experts model and included p‐tau181r and p‐tau217r in the predictive algorithm (Aβ42/40, Age and APOE) for amyloid PET status.

**Result:**

Plasma samples from N = 1085 composed of 67% Female, 13.5% Hispanic, 3.7% Black or African American with a mean age of 67.6 (SD = 6.1) years. 45% of participants had at least one APOE4 allele. The Area Under the Curve (AUC) for Aβ42/40 was 0.87 (95% CI; 0.84, 0.89), consistent with prior reports. For plasma tau, we found AUCs of 0.74 (95% CI; 0.71, 0.77) with p‐tau181, to 0.91 (95% CI; 0.90, 0.93) with p‐tau217r. The model including covariates p‐tau217r, Aβ42/40, Age and APOE improved AUC to 0.95 (95% CI; 0.93, 0.96).

**Conclusion:**

Our data suggests that plasma p‐tau217r in addition to Aβ42/40 ratio can significantly improve anti‐amyloid clinical trial screening burden and timelines for recruitment.

